# Supplementary material for: Imputation of Missing Values for Multi-Biospecimen Metabolomics Studies: Bias and Effects on Statistical Validity
Source: Metabolites. 2022 Jul 21;12(7):671. doi: 10.3390/metabo12070671 (PMC9317643; doi:10.3390/metabo12070671)
Supplement: Supplementary file 1 [file metabolites-12-00671-s001.zip › metabolites-1779817-supplementary.pdf]

**Supplementary Table S1:** Mean Correlation (std) between between-biospecimen Correlations of Real and Imputed Data for GCTOF data set by imputation method and approach (separate or combined matrices for imputation) based on 1000 simulated data sets.*

| Missingness | Approach | Half Min | KNN | RF | QRILC | EMB |
|---|---|---|---|---|---|---|
| 1% | Separate | 0.993 ± 0.001 | 0.953 ± 0.005 | 0.959 ± 0.004 | 0.982 ± 0.003 | 0.955 ± 0.006 |
| 1% | Combined | 0.993 ± 0.001 | 0.952 ± 0.005 | 0.954 ± 0.005 | 0.98 ± 0.003 | 0.951 ± 0.006 |
| 5% | Separate | 0.95 ± 0.005 | 0.863 ± 0.011 | 0.881 ± 0.01 | 0.919 ± 0.009 | 0.854 ± 0.013 |
| 5% | Combined | 0.95 ± 0.005 | 0.86 ± 0.011 | 0.867 ± 0.011 | 0.916 ± 0.01 | 0.843 ± 0.013 |
| 10% | Separate | 0.885 ± 0.012 | 0.802 ± 0.015 | 0.829 ± 0.012 | 0.835 ± 0.018 | 0.769 ± 0.02 |
| 10% | Combined | 0.885 ± 0.012 | 0.796 ± 0.015 | 0.815 ± 0.014 | 0.829 ± 0.016 | 0.751 ± 0.024 |
| 20% | Separate | 0.692 ± 0.026 | 0.748 ± 0.021 | 0.79 ± 0.018 | 0.61 ± 0.031 | 0.648 ± 0.031 |
| 20% | Combined | 0.692 ± 0.026 | 0.741 ± 0.023 | 0.769 ± 0.018 | 0.604 ± 0.034 | 0.636 ± 0.03 |
| 30% | Separate | 0.565 ± 0.037 | 0.638 ± 0.029 | 0.705 ± 0.021 | 0.45 ± 0.042 | 0.515 ± 0.037 |
| 30% | Combined | 0.565 ± 0.037 | 0.624 ± 0.033 | 0.67 ± 0.025 | 0.447 ± 0.042 | 0.504 ± 0.034 |
| 40% | Separate | 0.457 ± 0.038 | 0.506 ± 0.034 | 0.631 ± 0.03 | 0.33 ± 0.046 | 0.415 ± 0.042 |
| 40% | Combined | 0.457 ± 0.038 | 0.486 ± 0.037 | 0.586 ± 0.032 | 0.325 ± 0.051 | 0.407 ± 0.042 |
| 50% | Separate | 0.339 ± 0.045 | 0.371 ± 0.046 | 0.559 ± 0.034 | 0.199 ± 0.059 | 0.323 ± 0.049 |
| 50% | Combined | 0.339 ± 0.045 | 0.366 ± 0.045 | 0.498 ± 0.04 | 0.202 ± 0.052 | 0.322 ± 0.049 |
| 60% | Separate | 0.19 ± 0.046 | 0.23 ± 0.054 | 0.457 ± 0.033 | 0.089 ± 0.065 | 0.241 ± 0.049 |
| 60% | Combined | 0.19 ± 0.046 | 0.216 ± 0.055 | 0.379 ± 0.041 | 0.096 ± 0.053 | 0.234 ± 0.053 |

* HM: half the minimum observed value; kNN: k nearest neighbor; RF: random forest; QRILC: quantile regression; EMB: estimation/maximization with bootstrap.

**Supplementary Table S2.** Mean Correlation (std) between between-biospecimen Correlations of Real and Imputed Data for HILIC data set by imputation method and approach (separate or combined matrices for imputation) based on 1000 simulated data sets.*

| Missingness | Setting | Half Min | KNN | RF | QRILC | EMB |
|---|---|---|---|---|---|---|
| 1% | Separate | 0.995 ± 0.001 | 0.993 ± 0.002 | 0.994 ± 0.001 | 0.977 ± 0.003 | 0.993 ± 0.002 |
| 1% | Combined | 0.995 ± 0.001 | 0.994 ± 0.002 | 0.993 ± 0.002 | 0.976 ± 0.003 | 0.993 ± 0.002 |
| 5% | Separate | 0.973 ± 0.003 | 0.982 ± 0.004 | 0.984 ± 0.003 | 0.943 ± 0.006 | 0.977 ± 0.004 |
| 5% | Combined | 0.973 ± 0.003 | 0.981 ± 0.003 | 0.983 ± 0.003 | 0.942 ± 0.005 | 0.978 ± 0.005 |
| 10% | Separate | 0.952 ± 0.006 | 0.967 ± 0.005 | 0.972 ± 0.004 | 0.913 ± 0.009 | 0.954 ± 0.007 |
| 10% | Combined | 0.952 ± 0.006 | 0.962 ± 0.006 | 0.971 ± 0.005 | 0.912 ± 0.008 | 0.961 ± 0.007 |
| 20% | Separate | 0.879 ± 0.01 | 0.936 ± 0.007 | 0.948 ± 0.006 | 0.793 ± 0.015 | 0.909 ± 0.012 |
| 20% | Combined | 0.879 ± 0.01 | 0.935 ± 0.008 | 0.948 ± 0.007 | 0.793 ± 0.015 | 0.927 ± 0.01 |
| 30% | Separate | 0.826 ± 0.011 | 0.884 ± 0.011 | 0.915 ± 0.009 | 0.717 ± 0.018 | 0.845 ± 0.014 |
| 30% | Combined | 0.826 ± 0.011 | 0.895 ± 0.014 | 0.916 ± 0.01 | 0.714 ± 0.016 | 0.88 ± 0.013 |
| 40% | Separate | 0.77 ± 0.015 | 0.795 ± 0.021 | 0.863 ± 0.015 | 0.647 ± 0.021 | 0.764 ± 0.022 |
| 40% | Combined | 0.77 ± 0.015 | 0.835 ± 0.024 | 0.867 ± 0.015 | 0.647 ± 0.019 | 0.812 ± 0.024 |
| 50% | Separate | 0.675 ± 0.02 | 0.679 ± 0.023 | 0.787 ± 0.02 | 0.54 ± 0.025 | 0.662 ± 0.028 |
| 50% | Combined | 0.675 ± 0.02 | 0.727 ± 0.027 | 0.799 ± 0.018 | 0.539 ± 0.026 | 0.724 ± 0.027 |
| 60% | Separate | 0.54 ± 0.023 | 0.549 ± 0.025 | 0.668 ± 0.024 | 0.416 ± 0.028 | 0.544 ± 0.037 |
| 60% | Combined | 0.54 ± 0.023 | 0.598 ± 0.033 | 0.685 ± 0.024 | 0.422 ± 0.03 | 0.598 ± 0.037 |

* HM: half the minimum observed value; kNN: k nearest neighbor; RF: random forest; QRILC: quantile regression; EMB: estimation/maximization with bootstrap.
